# Pre‐exposure prophylaxis for men who have sex with men in China: challenges for routine implementation

**DOI:** 10.1002/jia2.25166

**Published:** 2018-07-12

**Authors:** Chongyi Wei, H Fisher Raymond

**Affiliations:** ^1^ Department of Social and Behavioral Health Sciences Rutgers School of Public Health Piscataway NJ USA; ^2^ Department of Epidemiology Rutgers School of Public Health Piscataway NJ USA

**Keywords:** PrEP, MSM (men who have sex with men), HIV/AIDS, China, prevention

In China, men who have sex with men (MSM) account for over a third and growing new HIV infections annually and is the only key population with increasing new infections 1, 2. A meta‐analysis of HIV incidence studies of Chinese MSM reported a pooled incidence of 5.61/100 person years, with an increasing trend over time 1. At present, HIV prevention efforts primarily focus on diagnosing new cases through expanded HIV testing services (e.g. rapid testing at MSM venues, distribution of HIV self‐testing kits by community‐based organizations) and initiating treatment immediately for those who are diagnosed with HIV. However, among HIV‐uninfected men, HIV testing uptake remains sub‐optimal 3.

Pre‐exposure prophylaxis (PrEP) is a highly efficacious HIV prevention method for individuals at risk for HIV infection 4. PrEP promotion, academic detailing for providers (i.e. face‐to‐face education of prescribers to improve prescribing of drugs to be consistent with medical evidence and clinical guidelines), and social marketing campaigns have been rolled out across the US to raise awareness. While PrEP has re‐energized our hope of ending the HIV epidemic as part of a combination prevention and treatment package, its eventual impact on reducing new infections at the population‐level will depend on routine implementation and uptake in the real world. PrEP as an intervention may be particularly challenging among key populations such as MSM in low‐ and middle‐income countries where socio‐cultural contexts and healthcare infrastructure is vastly different from that in developed countries. China provides an example where implementing PrEP among MSM faces considerable unique challenges.

There are long held cultural beliefs in China that good health is promoted through exercise, a balanced diet and good relations with family and friends but when illness *occurs,* addressing the problem through traditional medicine is preferred as western medicine overly focuses on symptoms rather than the cause of the illness 5, 6. Nowhere in these traditions are beliefs that taking medicine *before* illness occurs, such as in the case of PrEP, is desirable. Therefore, HIV‐uninfected MSM may be reluctant to take ARV drugs as a prevention option. In fact, several cross‐sectional surveys reported low to moderate acceptability of daily PrEP use among Chinese MSM 7, 8, 9, 10, and an intervention trial conducted among over 1,000 MSM in Shanghai reported that only 19% of participants expressed willingness to use tenofovirdisoproxil fumarate (TDF) on a daily basis at baseline and a mere 3% actually participated in the TDF group and took one tablet a day 11. A follow‐up qualitative evaluation of the trial identified that concerns of side effects was one of the main reasons for not wanting to use PrEP 12. Another study conducted among MSM in Hong Kong found that actual uptake of PrEP was only 1% 8. Furthermore, recent scandals surrounding powdered milk and vaccines have fuelled growing concerns over food and drug safety among the general public.

Despite nation‐wide anti‐HIV stigma campaigns and the growing visibility of LGBT populations, HIV stigma and homophobia still persist and are widespread. A majority of Chinese gay and bisexual men and other MSM do not disclose their sexual orientation or same‐sex behaviour to their families or healthcare providers. As a result, many do not access HIV prevention and care services, such as HIV testing, in fear that their sexual minority status would be exposed to others 13. In addition to sexual minority‐related stigma, HIV‐related stigma and discrimination still persist within MSM communities and among the general public. Thus, HIV‐uninfected MSM on PrEP may risk “outing” themselves or having others suspect that they are leaving with HIV 14, 15.

At the community‐level, there is a severe lack of awareness and correct knowledge of the benefits and effectiveness of ART, even among MSM living with HIV 16, 17, 18. For example, many do not know that HIV is a manageable disease, equate it to AIDS or death, and have concerns with treatment 16. HIV‐related information dissemination among MSM communities has also not kept up with advances in the field. A couple recently published studies reported that just 17% and 34% of Chinese MSM had ever heard of PrEP respectively 7, 19. These are in part due to a weak civil society and the lack of resource‐rich community‐based organizations that serve the health needs of MSM populations as a result of government regulations that limit international funding to such organizations and a hostile environment for LGBT groups. This lack of support for LGBT civil society leads to low levels of community engagement, MSM communities as a whole and community‐based organizations in particular, which is essential to raising PrEP awareness.

Moreover, wide‐scale implementation of PrEP will not be feasible without structural‐level changes of the Chinese healthcare system. At present, the ways in which healthcare services are provided to individual patients and certain prescription medications are covered by health insurance do not encourage uptake of PrEP at the individual‐level or fully support its scale‐up at the population‐level. Unlike in more developed countries, most Chinese people do not have their own family providers, not to mention the scarcity of physicians who specialize in HIV care or have expertise in sexual health or gay men's health. This begs the question of how education, counselling, risk assessment and tailoring, and on‐going monitoring can be accurately and effectively delivered to MSM who might be interested in PrEP. Compounding this significant barrier, ARV drugs are currently not accessible or affordable to HIV‐uninfected individuals. To improve access to treatment, the government has long been providing free generic ARV drugs to people living with HIV through designated hospitals under the “Four Frees, One Care” initiative, but this is likely not a sustainable approach if it were expanded to millions of MSM at high risk for HIV infection. A person living with HIV can also purchase much pricier imported drugs at private pharmacies, but the expense is entirely out of pocket as ARV drugs are not listed prescriptions covered under private health insurance.

The Chinese context suggests that there are myriad barriers and challenges to routine PrEP implementation and scale‐up among MSM in China. Successful PrEP rollout will first depend on establishing an equitable community planning process among stakeholders where health authorities should more fully engage and support community‐based organizations. Second, broader campaigns that address negative beliefs and lack of knowledge around medications' safety and efficacy as well as HIV and sexual minority stigma should be launched during rollout. Third, PrEP should be made available through government and community clinics where MSM community partners can play a role in supporting counselling and monitoring. Finally, guidelines and policies should be implemented to protect the health rights of LGBT groups and ensure equal access to PrEP regardless of sexual minority status. Without systematically addressing these challenges, PrEP will not reach its prevention potential and make a meaningful contribution to curtailing the HIV epidemic among Chinese MSM.

## Competing interests

There are no competing interests.

## Authors' contributions

CW and HFR co‐wrote this paper.
